# An unusual complication of silver nitrate therapy for chyluria

**DOI:** 10.4103/0970-1591.32078

**Published:** 2007

**Authors:** Jayesh V. Dhabalia, Girish G. Nelivigi, Manav Suryavanshi, Shal Kakkattil, Vikash Kumar Singh

**Affiliations:** Department of Urology, Seth GS Medical College and KEM Hospital, Mumbai, India

**Keywords:** Chyluria, pseudoaneurysm, silver nitrate

## Abstract

Chyluria is a common disorder in the tropics. In our country it is most commonly managed by instillation of silver nitrate. We report a patient who developed severe perinephric hematoma due to pseudoaneurysm of renal artery following silver nitrate instillation. He was managed by angioembolisation followed by drainage of infected perinephric hematoma. We discuss various modalities of treatment of chyluria including complications of silver nitrate therapy.

## INTRODUCTION

Chyluria is a common problem in India. It is commonly treated by endoscopic instillation of silver nitrate. Despite long history of its use, there are many unresolved issues like the dosage, concentration and duration of therapy. More importantly there are serious safety concerns which are highlighted by the many case reports in urological literature reporting severe morbidity and even mortality after silver nitrate instillation. We report one such patient who experienced a major complication following this treatment and also discuss the complications of silver nitrate instillation reported in literature.

## CASE REPORT

A 30-year-old male presented with chyluria of filarial origin. He underwent cystoscopic instillation of 1% silver nitrate in the right kidney. The ureteric catheter was introduced up to the lower ureter and 7cc of silver nitrate was instilled without use of excessive pressure. Within a few hours after the procedure the patient developed distension of abdomen, oliguria and hematuria. On examination there was a 15 × 15 cm size tender lump in right flank. Ultrasonography and CT scan of abdomen showed a large perinephric hematoma measuring 8 × 10 cm on the right side with normal contrast uptake in the kidney. Hemoglobin decreased from 13 gm% before the procedure to 9 gm% after.

**Figure 1:**
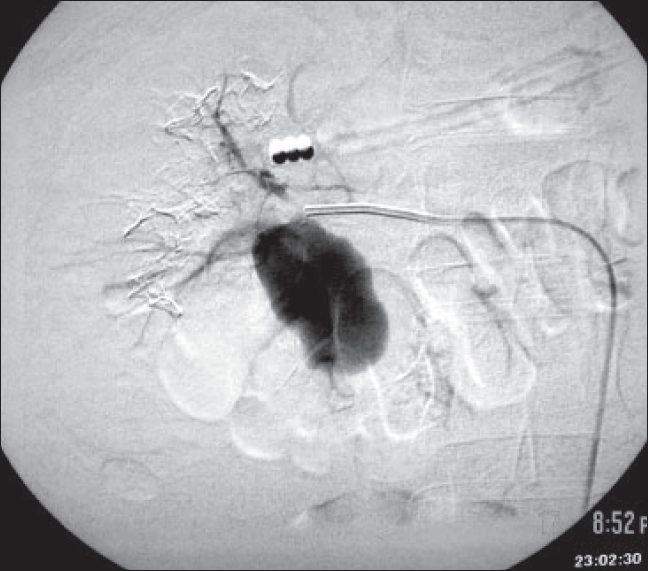
pseudoaneurysm of renal artery

**Figure 2:**
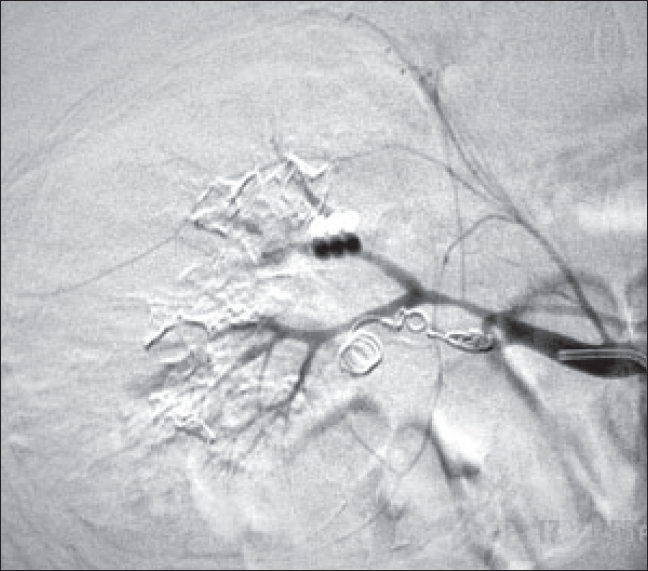
Post angioembolisation

In view of progressively increasing abdominal distension, persistent hematuria and drop in hemoglobin, the patient was subjected to renal angiography. It showed a pseudoaneurysm in postero-inferior branch of the right renal artery [Figure 1]. Coil embolization was then done after which the patient stabilized [Figure 2]. Two days after the procedure, the patient developed high fever and leucocytosis of 23000/cc. Repeat CT scan showed infected perinephric hematoma on the right side with abscess. He subsequently underwent incision and drainage of the abscess. The patient was discharged after complete resolution of infection.

On follow-up, the patient is asymptomatic with normal serum creatinine.

## DISCUSSION

Various modalities of treatment of filarial chyluria like retroperitoneal lymphatics - spermatic veins shunt, renal decapsulation and lymphatic disconnection at the renal hilum have been described.

Silver nitrate therapy was first described in 1964 and has been the most popular form of treatment of chyluria. It is a sclerosant leading to fibrotic occlusion of pyelolymphatic channels when injected into the collecting system of the kidney. Reports in the literature have validated the safety and efficacy of silver nitrate therapy. However, many serious complications following silver nitrate therapy have been reported from India and abroad.

One of the earliest complications was in a case of hematuria. The patient developed anuria following intravesical instillation of silver nitrate.

Mandhani *et al* reported death following complications of bilateral silver nitrate therapy for chyluria.[1] Other complications like pelvi-calyceal cast formation; argyrosis of urinary tract, acute renal failure and renal papillary necrosis after silver nitrate therapy have also been reported.[2]

However, only one case of renal artery pseudoaneurysm as a complication of silver nitrate installation has been reported. The patient was treated successfully by coil embolization.[3] The mechanism by which silver nitrate instillation results in pseudoaneurysm formation is not known. In our case it is possible that there was leakage of silver nitrate outside the pelvis with chemical injury to renal artery resulting in pseudoaneurysm formation. Infection resulting in pseudoaneurysm formation is another possibility but is less likely as the patient developed flank mass within hours of instillation. Trauma is a common cause of pseudoaneurysm. However, in this case it could not have been the cause as the catheter was kept in the lower ureter during instillation.

The purpose of this case report is to bring into focus the safety issues concerned with silver nitrate therapy. The safety profile of silver nitrate has never been established in animal experiments. The dose to be administered in terms of quantity and duration has also not been standardized. The therapeutic concentration of silver nitrate too is undetermined ranging from 1-3% in various reported studies. In view of these facts and the fact that an equally efficacious and less harmful treatment like Betadine is freely available, a consensus on the usage of silver nitrate in the human body based on scientific evidence is needed.
